# Perceived Academic Self-Efficacy among Romanian Upper Secondary Education Students

**DOI:** 10.3390/ijerph17134689

**Published:** 2020-06-29

**Authors:** Ana-Maria Zamfir, Cristina Mocanu

**Affiliations:** National Scientific Research Institute for Labour and Social Protection, 6-8 Povernei Street, 010643 Bucharest, Romania; mocanu@incsmps.ro

**Keywords:** self-efficacy, test anxiety, academic performances, baccalaureate exam, upper secondary education

## Abstract

Perceived academic self-efficacy represents an important component of students’ mental health and well-being. The link between efficacy beliefs and level of academic performances relies on the fact that they regulate the anxiety and foster motivation, school engagement, effort, and perseverance of students. This paper aims to identify factors that are conducive for more efficacious beliefs in different socio-economic and educational contexts. We build our analysis on data collected from a sample of Romanian upper secondary education students on their beliefs in relation to their ability to perform at the baccalaureate exam. We employ decision tree models in order to unveil the way factors interact and predict perceived academic self-efficacy, with focus on the positive support received from parents and teachers, as well as on features of the school environment. Our results can be useful for building more resilient educational environments that support mental health and academic well-being of students.

## 1. Introduction

Self-efficacy is a key concept introduced by the Social Cognitive Theory referring to peoples’ beliefs in their personal ability to achieve the desired results [1]. Such beliefs are important for self-knowledge and play a key role in the process of personal agency [1,2,3]. Many scholars have studied the importance of self-efficacy in the educational field [4,5], especially in relation to academic performance [6,7,8]. They have been interested in analyzing the mechanisms that explain the way self-efficacy influences academic performances, as well as educational and career choices.

Perceived self-efficacy is crucial for the engagement, effort, and perseverance in learning and, as a result, those with lower efficacious beliefs are vulnerable to follow a negative spiral in which low expectations lead to less effort, lower success, and lower self-efficacy beliefs. Previous findings have demonstrated that self-efficacy influences the mental health as those with low efficacy are more vulnerable to anxiety, worry, depression, and social avoidance [9]. Those with poor confidence in their capabilities consider difficult tasks as threats, have weak ability to cope with pressure, become stressed or depressed, and display higher probability to give up [10]. This relation has also been confirmed in the case of adolescents [11,12]. Given these arguments, some scholars consider the perceived self-efficacy as a cognitive precursor of anxiety and depression [13] as those who do not believe that they are capable to cope with difficult tasks and challenges feel higher levels of anxiety [3]. In the education field, such challenges include academic tests and exams. Furthermore, previous research showed that self-efficiency is a significant predictor of test anxiety [14], influencing the academic well-being. Higher levels of anxiety negatively affect academic effort and motivation, reducing performances.

Considering the importance of academic self-efficacy for the mental health and academic well-being of students, our paper aims to identify factors that shape perceived self-efficacy in relation to the baccalaureate exam in different socio-economic and educational contexts. We investigate beliefs of Romanian upper secondary education students with respect to the performances they believe they are capable to achieve at the baccalaureate exam. In the Romanian education system, performances obtained by students at the baccalaureate exam are very important as they condition the admission in higher education. Being considered an important and difficult exam at the end of upper secondary education cycle that influences the chances of entering in tertiary education and shapes the access to various educational opportunities, this exam is seen as a key challenge for Romanian students.

## 2. Literature Review

Numerous scholars have showed the importance of self-efficacy for academic achievements. Some studies focused on specific academic fields, demonstrating, for example, that perceived self-efficacy in school mathematics or English influences academic performances, irrespective of the level of ability in mathematics or English, respectively. In fact, it has been demonstrated that perceived self-efficacy predicts more accurate academic performance than the ability level itself [15,16]. Other classes of studies have focused on gender differences in perceived self-efficacy, demonstrating variation of these differences in relation with the age and the domain of study [17,18]. Self-efficacy differences explain part of the educational preferences and choices leading to underrepresentation of genders in various fields. From this point of view, one can say that perceived self-efficacy can influence the course of lives [18].

Perceived self-efficacy shapes educational and career choices, motivation, effort of the students, and their response in face of adversity [1,19]. Self-efficacy influences the effort and perseverance of students that shape the learning process and subsequently the obtained achievements [3,5,20,21]. The idea is that efficacy beliefs are not simply predictors of achievements as the presence of more efficacious beliefs triggers actions and perseverance in learning [20].

According to Bandura [3], there are four sources of experiences for self-efficacy beliefs: (1) Mastery experiences; (2) vicarious experiences; (3) verbal or social persuasion; and (4) psychological and emotional states. All of these sources were extensively analyzed by the scientists in the field in relation to academic perceived self-efficacy and achievements, as well as to reciprocal causality among them [22]. Bandura’s concept and theory are mainly focused on internal factors of how an individual interprets, accesses, modulates, integrates previous information and experiences, and understands and reacts to the new ones, while external factors are treated as having an indirect influence [23].

Although the importance of self-efficacy in predicting behaviors and achievements is extensively analyzed and confirmed by majority of studies, there have been several limits to its application pointed out. For instance, Bandura underlined that self-efficacy beliefs are conditioned by the social economic context and that they cannot be a substitute for any prejudice of educational system [1,22]. If schools lack basic resources, both financial and adequately qualified teachers, then self-efficacy will not perform its role in influencing and nurturing academic achievements.

Moreover, previous studies have showed that self-efficacy beliefs are rather stable in time [23,24]. It is expected that self-efficacy can be improved by exposure to successful experiences and social persuasion such as support, feedback, and encouragements from other people [25], but the malleability of self-efficacy is still a matter of debate [23]. Thus, understanding how self-efficacy is defined in childhood is even more important.

As previous experiences shape students’ confidence in their academic capabilities, having successful academic experiences that foster perceived self-efficiency is a way to change poor academic well-being [26]. However, the social-economic contexts in which studies on the influence of self-efficacy were carried out are pretty homogenous, focusing on how self-efficacy beliefs mediate for instance the influence of socio-economic background on academic achievements [27]. Thus, some critics aimed to examine how self-efficacy theory works in context of inequality, arguing that self-efficacy concept, as defined by Bandura [1], put too much responsibility on individuals and their agency, minimizing the fact that they do not have control on the structural inequalities in the redistribution of resources [28]. Socio-economic status impacts psychosocial processes and thus indirectly affects efficacy beliefs and educational aspirations and outcomes [29]. The quality of education, both in terms of infrastructure and resources, as well as its distribution in population influence the educational development. There are evidences provided by longitudinal approaches that level of cognitive skills developed through mandatory education can support high academic aspirations and achievements even when perceived self-efficacy and self-regulation register some fluctuations [29].

Self-efficacy is embedded in the environment in which a person lives [3]. Family is the most important context in which a child develops and learns. The importance of family involvement in education is extensively analyzed and confirmed by a large amount of studies aiming to explain school engagement and academic achievements or failure (including dropout).

However, studies aiming to link the two strands of research—self-efficacy and parental involvement in education—are rather new [30]. Some of the studies introduce the concept of parents’ self-efficacy beliefs in the equation aiming to put together both self-efficacy, socio-economic background, and academic achievements [27,31].

The four sources of self-efficacy postulated by the social cognitive theory acquire new features by analyzing and understanding the role of family (that could include grandparents and older siblings) in the process of child’s or adolescent’s development, including the development of self-efficacy [30,32].

Scientific literature extensively discussed the influence of different types of parents’ involvement in education on educational outcomes. Most of the studies on parental involvement in education and relation with self-efficacy address primary education, and even fewer the adolescence and the upper secondary education [33]. It is expected that parents’ involvement and its influence on academic achievements decreases with age, while it is expected that adolescents’ autonomy increases [27].

Parents provide financial, material, and psychological resources for children schooling [34]. They select and pay for private tutoring [35,36], they assist or help their children with homework, they select or support the children preferences for extra-curriculum activities, spend time with children, supervise the learning processes outside school, they are involved in different voluntary activities, are organized in communities or bodies and influence school decisions, they communicate with teachers and school, and they have aspirations and expectations with respect to their children academic achievements [27,37,38].

Coming back to the four sources of self-efficacy, there are authors redefining the role of parents and family in relation to all four sources. Family and more specifically the parents are implicit components in each source of self-efficacy. Through interactive processes, parents may support children process of understanding the new tasks and support the process of identifying previous similar situations, information, and ways to react and solve a specific problem [39]. So, they influence the mastery experience. Parents can model children’s experiences, make demonstrations, select the appropriate terms, individuals, and standards to perform a specific task [39]. They are certainly among the essential people that can contribute to the empowering of children, providing them the confidence that they have knowledge and skills to solve a specific task. Last but not least, the parent–children interaction in relation to education and school can produce negative or positive emotions, and so they influence the psychological state.

Therefore, much of the research on the impact of parents’ involvement in education provided mixed results [33,40], highlighting the relevance of the type of involvement and of the socio-economic background. Parents’ aspirations remain one of the strongest predictors for educational achievements in all meta-analyses [41,42]. Socio-economic status still explains, to a large extent, both children and parents’ educational aspirations and achievements [27], although their impact on educational outcomes are mediated by self-efficacy. Children from families with high socio-economic status seem to benefit more from parents’ different types of capital, while due to the lack of financial resources, parents and children from families with fewer resources undermine, to a certain extent, their self-efficacy [31]. In other words, self-efficacy provides better results for those better socially positioned and to a certain extent may be used for explaining persistent inequalities, even in countries that made efforts to equitably finance public schooling.

Studies on educational attainment among Romanian children and adolescents evidenced the strong influence of parental involvement [36,43,44], but also the role of school governance, school administration, teaching methods, and school and community characteristics [44,45]. PISA assessments evidenced, in all its waves carried out in Romania, both the high share of students with low level of skills, as well as the high inequalities in educational performances in relation to the socio-economic background [46]. Analyses on the role of different actors involved in educational process in Romania evidenced the crucial role of family support in disadvantaged communities, mainly in reducing absenteeism and drop out [47]. School dropout occurs from primary education until university and it is poorly addressed by programs and policies, even if its causes rooted in high poverty and low parents’ education and presence of materialistic values are extensively addressed by research focused on Romanian case [48,49].

Thus, we focus our study on the patterns of variation of perceived academic self-efficacy among upper secondary education students in the Romanian context, analyzing the influence of the support received from parents and teachers, as well as various features of the school environment. We hypothesized that the support of parents and teachers is positively associated with more efficacious beliefs. Also, we expected that characteristics of the school environment that can produce positive experiences for students at school will predict a higher perceived academic self-efficacy. Additionally, we expected that the outcome of previous challenging experiences will predict the level of self-efficacy. Finally, the study aims to analyze how the influence of the above stated predictors vary across various educational contexts in Romania.

## 3. Data and Methods

### 3.1. Participants

This paper aims to explore factors that interplay and predict perceived academic self-efficacy among upper secondary education students. We build our analysis on data collected in November 2019 from a sample of 502 students enrolled in the final grade of the high school. Surveyed students were enrolled in 35 different high schools in Romania and their average age was of 17.9 years old. Other socio-demographic characteristics are presented in Table 1, indicating a well-balanced sample.

### 3.2. Instruments

Data were collected via a questionnaire-based survey in the first months of the final grade of the upper secondary education cycle. Perceived academic self-efficacy was measured in relation to the baccalaureate exam. The item measuring perceived academic self-efficacy for the baccalaureate exam was as follows: “I believe I can obtain a final grade to baccalaureate exam of about: (1) <6.00, (2) 6.00–6.99, (3) 7.00–7.99, (4) 8.00–8.99, (5) 9.00–10.00”. Baccalaureate is the final exam that takes place at the end of final grade of high school. The final mark obtained by students to this exam (calculated as average of marks obtained to different discipline tests) is essential for their admission into higher education and for educational choices that will be available for them after completing upper secondary education. In the Romanian education system, marks vary between 1.00 and 10.00, where 10.00 is the maximum mark. In order for the baccalaureate exam to be passed, one should obtain a final grade of 6.00 or higher. Therefore, we build three classes for the dependent variable: (1) Grade less than 6.00, (2) grade of 6.00–7.99, and (3) grade of 8.00–10.00. Obtaining a grade less than 6.00 means that the student did not pass the baccalaureate exam and he/she cannot apply for admission into higher education. Moreover, in case of passing the exam, the mark obtained represents an important criterion for admission in tertiary education programs. So, we recoded the classes of the dependent variable in order to classify students in three groups: Students with poor level of perceived self-efficacy (who display failure beliefs), students with medium level of self-efficacy, and students with high efficacious beliefs.

Predictors included in the analysis cover aspects related to the availability of the support from parents and teachers as it is perceived by the students as well as prevalence of various perceived features of the school environment: Satisfactory extracurricular activities; buildings, facilities and sport infrastructure of quality; opportunity for the development of personal abilities and talents. The support received from parents and teachers has been measured by asking the students a multiple response question: “In preparing for the baccalaureate exam, I rely on the support of…: (1) my parents, (2) my teachers, (3) other persons, (4) none of the above”. On the base of this item, we constructed two dummy variables for the support of parents and teachers, taking the values 1 = ”yes” and 0 = ”no”. For assessing the various features of the school environment, the students were asked to rate the presence of each considered aspect (satisfactory extracurricular activities; buildings, facilities and sport infrastructure of quality; opportunity for the development of personal abilities and talents) in relation with their school environment on a scale ranging from 1 = ”very low extent” to 4 = ”very high extent”. We recoded the first two categories with the value 0 = ”less extent” and the last two categories with the value 1 = ”high extent”. Gender of the students was also taken into account as independent variable.

Additionally, we included in the analysis one variable measuring a hypothetical choice of the students for the preferred high school and educational profile they would like to be enrolled in, with 4 classes: (1) I would choose the same high school and the same educational profile as the ones in which I am currently enrolled in; (2) I would choose the same high school, but a different educational profile as the ones in which I am currently enrolled in; (3) I would choose a different high school and the same educational profile as the ones in which I am currently enrolled in; (4) I would choose a different high school and a different educational profile as the ones in which I am currently enrolled in. This variable represents both an indicator of the general satisfaction regarding the educational environment, as well as an indicator of students’ feelings regarding the outcome of a previous challenging experience. In Romania, the admission in high schools and educational profiles is a selection process based on the hierarchy of individual preferences of students, conditioned by their average grade calculated from marks obtained in lower secondary education cycle (20%) and marks obtained at the “capacitate” exam (80%).

Accuracy of the variables included as predictors in the analysis was checked by bootstrapping technique based on stratified random sampling. The stratified random sampling was performed on the base of the same variables of stratification by which the original sample has been stratified, namely the educational profile (general, vocational) and area of residence (urban, rural) of the students. We performed bootstrapping based on stratified sampling as it is more powerful than the one based on simple random sampling. The bias was near zero in every instance, confirming the accuracy. Descriptive statistics and bootstrap estimates for percent for all the predictors are provided in Table A1 from Appendix A.

### 3.3. Statistical Analysis

In order to shed light on the main factors predicting perceived academic self-efficacy, we employed decision tree models that are able to unveil patterns in data, producing tree-based classification models that group students based on a set of factors with respect of their likelihood to belong to one of the classes of the dependent variable [50,51]. Tree-based algorithms are very powerful predictive models with high performances of accuracy, stability, and ease of interpretation. They are highly performant for complex, non-linear relationships in data. Moreover, the tree-based models are ideal for capturing interactions between independent variables. Outputs of tree-based classification models highlight groups and sub-groups of homogenous cases that are called parent and child nodes of the tree. The model predicts the most commonly occurring class in each node (sub-group of cases). So, one of the main advantages of tree-based models relies on the fact that they allow for identification of distinct sub-groups of cases based on the interaction of significant independent variables for which predicted values are calculated. Moreover, in the case of tree-based models, one independent variable might be used for more than one split of the tree or not at all, which allows the identification of complex interaction relations. For the development of the decision tree models, we used the Chi-squared automatic interaction detection (CHAID) growing method. In each step of the growing tree, the model identifies the independent variable that has the strongest interaction with the dependent one. Significance level for splitting nodes in the models has been of 0.05. Also, in the case of categorical independent variables, the model can merge categories that have a similar pattern of association with the dependent variable.

Furthermore, as performances registered at baccalaureate exam display significant inequalities, different socio-economic and educational contexts were considered for developing different decision tree models in order to identify similarities and variations in the manifestations of perceived academic self-efficacy.

## 4. Results

Our results show that 12.6% of the surveyed students have failure beliefs in relation to the exam that they are going to take at the end of the school year (grades less than 6.00), while 35.2% students display medium levels of self-efficacy (6.00–7.99) and 52.2% believe that they are able to obtain high grades at baccalaureate exam (8.00–10.00).

**Figure 1:**
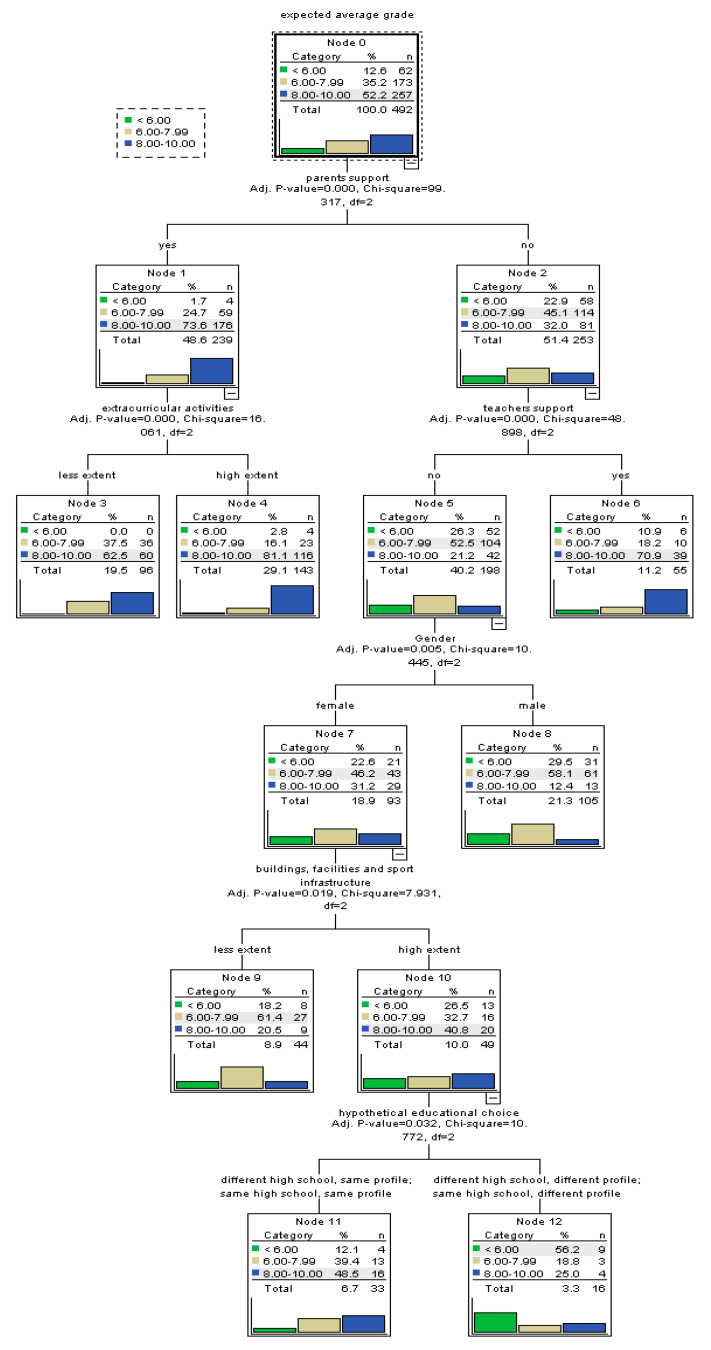
Tree-based model for perceived self-efficacy at baccalaureate exam.

The first tree-based model has been constructed for the entire sample of students in order to identify factors that predict efficacious beliefs with respect to the baccalaureate exam (Figure 1). We found six significant predictors for perceived self-efficacy. The final model comprises 13 nodes, out of which 7 are final nodes. Out of all factors, the strongest predictor for self-efficacy is the perceived support received from parents. It is positively associated with more efficacious beliefs. Additionally, for students relying on their parents’ support, participation to satisfactory extracurricular activities in the school environment represents a further predictor of high self-efficacy. Students with the support of their parents and benefiting from extracurricular activities display the highest probability of having high efficacious beliefs (81.1%). On the other hand, the tree-based model has grown more from the node of students with no perceived support from their parents. Thus, for these students, the perceived support from their teachers is the next important predictor positively associated with self-efficacy. Furthermore, for those lacking the perceived support from parents and teachers, another significant predictor is the gender, with female students displaying higher probabilities of more efficacious beliefs than males. In the case of this sub-group of female students, the existence of buildings, facilities, and sport infrastructure of quality in the school environment is associated with higher self-efficacy. Finally, for female students lacking the support from parents and teachers and who benefit from quality school infrastructure, satisfaction with the educational profile in which they are enrolled in predicts self-efficacy in relation with the next exam. Those who would choose the same profile as that they are currently enrolled in display more efficacious beliefs, while those who would choose a different educational profile have the highest probability of failure beliefs (56.2%).

**Figure 2:**
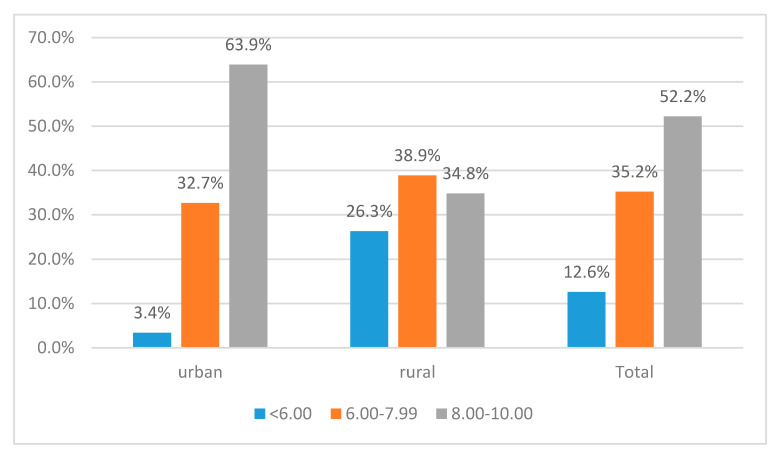
Perceived self-efficacy at baccalaureate exam by area of residence.

Perceived self-efficacy varies significantly by area of residence (Figure 2). The significance of the relation has been confirmed by the chi-square test (*p* = 0.000). Failure beliefs are significantly more prevalent in rural areas as 26.3% of rural students do not feel able to obtain a final grade above 6.00 at the baccalaureate exam, as compared with 3.4% in urban areas. Also, urban areas register a significantly higher concentration of those with high self-efficacy (63.9%) than rural areas (34.8%). Taking this result into account, we constructed different tree-based models for urban and rural students.

**Figure 3:**
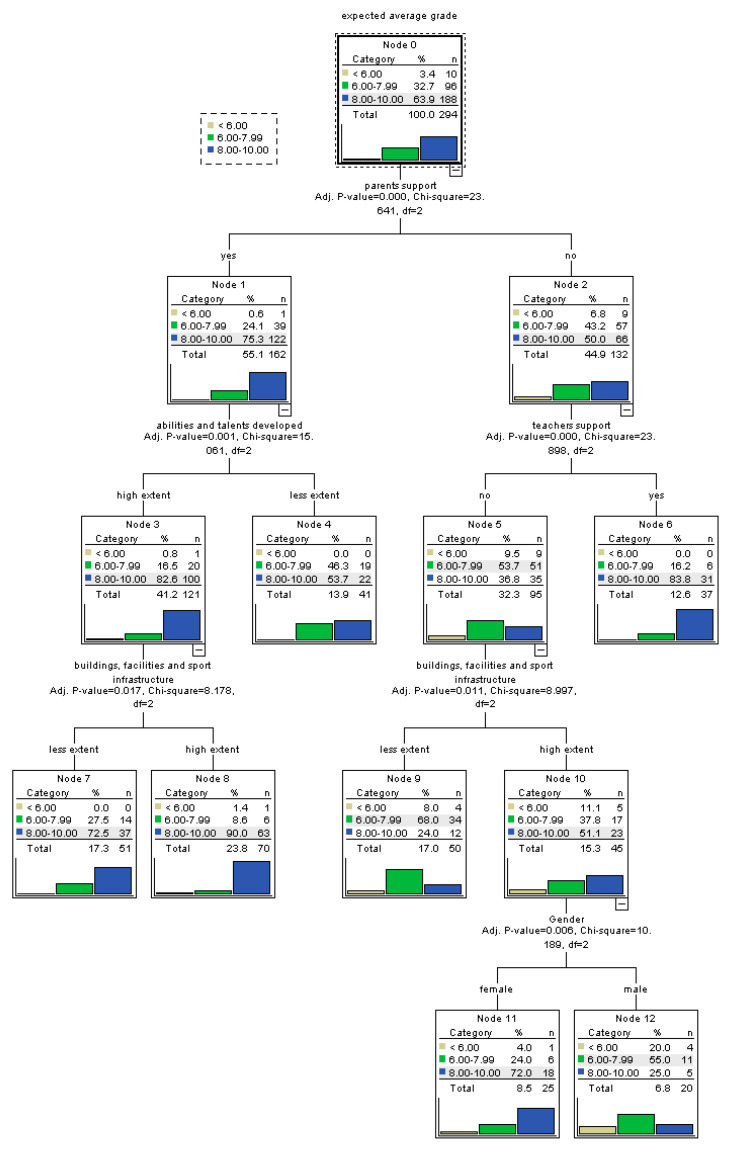
Tree-based model for perceived self-efficacy at baccalaureate exam among urban students.

For the sub-sample of urban students (Figure 3), perceived parents support is again the strongest predictor for self-efficacy. For urban students who feel supported by their parents, a further predictor is the belief that their abilities and talents are being developed at school. Those who consider that their personal abilities and talents are been developed have higher probability of more efficacious beliefs. Moreover, for them, the perceived existence of buildings, facilities, and sport infrastructure of quality further improves the probability of having high self-efficacy. In the case of urban students lacking the support of parents, an important predictor is the perceived support of teachers that significantly improves the probability of efficacious beliefs. For students who feel that they do not have the support of parents or teachers, the perceived quality of buildings, facilities, and sport infrastructure increases the probability of efficacious beliefs. For those lacking the support, but who feel that they benefit from a school infrastructure of quality, gender represents a further factor of influence as female students display significant higher probability of more efficacious beliefs.

**Figure 4:**
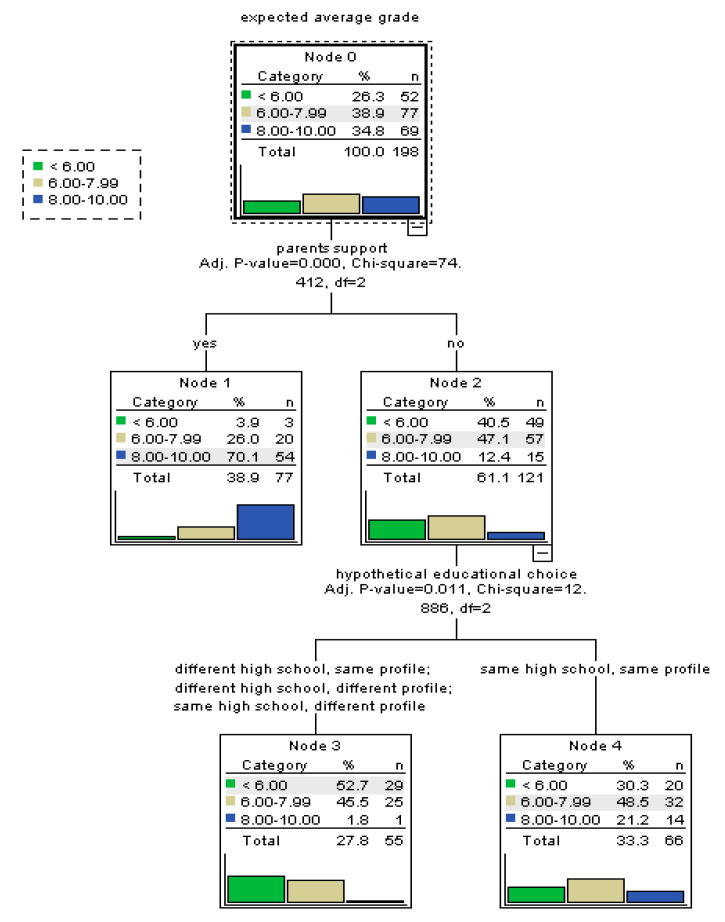
Tree-based model for perceived self-efficacy at baccalaureate exam among rural students.

For the sub-sample of rural students (Figure 4), the constructed tree-based model retained fewer significant predictors. Perceived support of parents remains the most important factor. The availability of parents’ support increases the probability of more efficacious beliefs in a significant extent, while lack of the support is associated with less efficacious beliefs. Moreover, for rural students who do not feel that they have their parents support, the satisfaction with the high school and educational profile in which they are enrolled in represents a further factor of influence. Thus, rural students lacking the parents’ support who would prefer a different high school or educational profile than the ones in which they are currently enrolled in present the highest probability of poor perceived self-efficacy.

**Figure 5:**
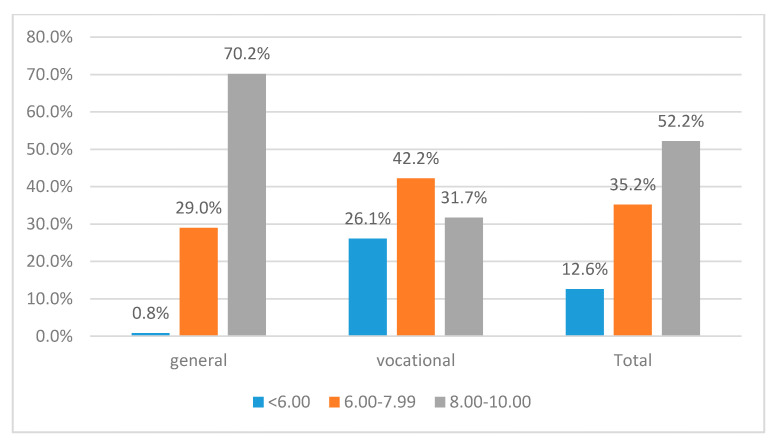
Perceived self-efficacy at baccalaureate exam by type of educational program.

On the other hand, our results show that there is a significant association between perceived self-efficacy and the type of educational track (*p* = 0.000). Students enrolled in general education have more efficacious beliefs, while students enrolled in vocational education display lower levels of self-efficacy (Figure 5).

**Figure 6:**
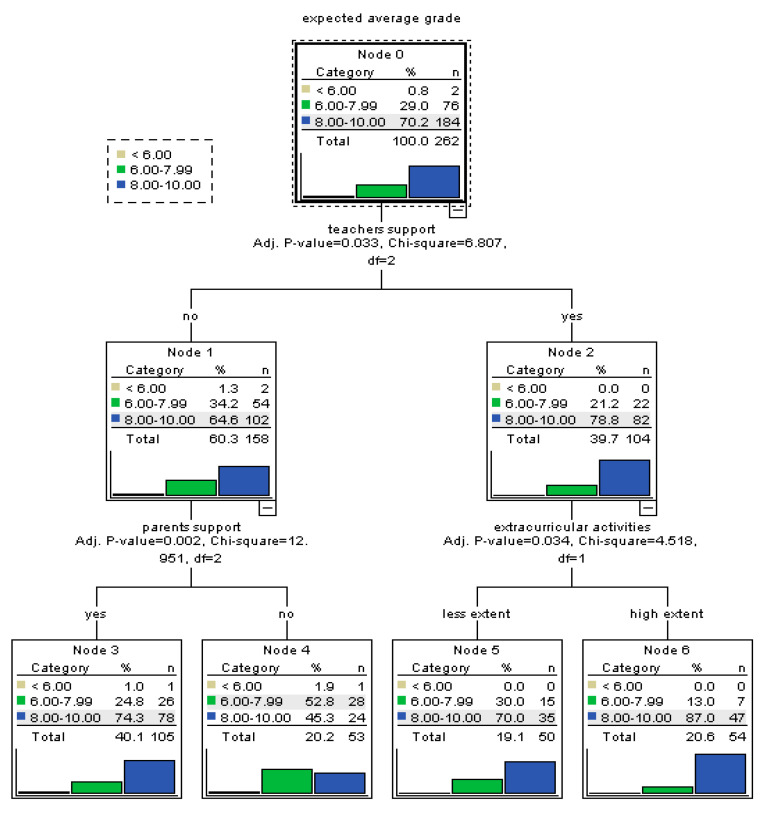
Tree-based model for perceived self-efficacy at baccalaureate exam among students enrolled in general education.

In the case of students enrolled in general education, the support provided by teachers represents the most important predictor for self-efficacy. Those enjoying the support of teachers display more efficacious beliefs than the others. Still, for students who feel they do not rely on the teachers’ support, the perceived support from parents is conducive for self-efficacy. Furthermore, in the case of students enrolled in general educational who rely on their teachers’ support, the existence of satisfactory extracurricular activities improves academic self-efficacy (Figure 6).

**Figure 7:**
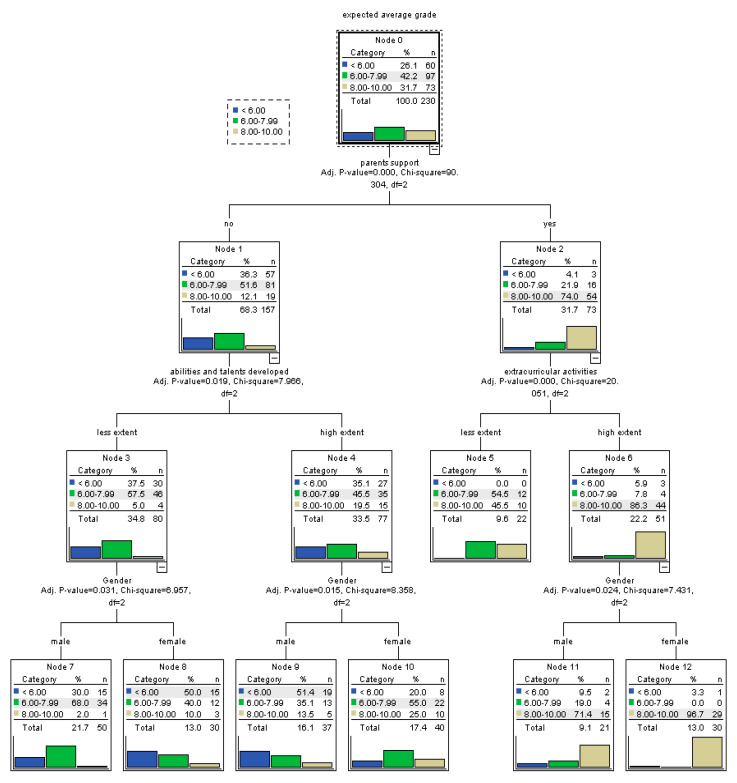
Tree-based model for perceived self-efficacy at baccalaureate exam among students enrolled in vocational education.

In the case of vocational education students, the strongest predictor for self-efficacy is, again, the parents’ support. Students relying on their family support have much higher probabilities of high self-efficacy. Moreover, for these students, the participation in extracurricular activities can further improve self-efficacy, especially among females. On the other hand, for vocational students lacking the support of parents, the opportunity to develop their abilities and talents at school improves self-efficacy. In the case of those benefiting from abilities and talents development, it is more probable for females to have more efficacious belies, while in the case of those who do not feel that their abilities and talents are being developed at school, females display high probability of failure beliefs (Figure 7).

## 5. Discussion

Our results show that around 13% of Romanian students do not feel capable to pass the baccalaureate exam. From the mental health and well-being point of view, they can be vulnerable to test anxiety and depression, which can reduce their motivation, effort, and final performance. Our findings are consistent with previous studies [30,32,33,39], showing that the support of parents is a central factor conducive for more efficacious beliefs among students in all the analyzed contexts. Students who consider that they are supported by their parents in educational matters display higher probability of more efficacious beliefs. Therefore, our results confirm the role of parents support for self-efficacy, including in the case of adolescents who can be considered more autonomous than younger children [27].

The support of teachers is also very important for enhancing the perceived self-efficacy, especially for students lacking the support of parents. Students with no support from parents but benefitting from teachers support also register a high probability of more efficacious beliefs. Still, it seems that the role of teachers for self-efficacy is not significant for students in rural areas and vocational programs. On the other hand, support of teachers is the strongest predictor for the self-efficacy of students enrolled in general education. This result could be linked to inequalities embedded in the Romanian educational system in terms of resources allocation and availability of qualified teachers. The importance of parents and teachers support for perceived academic self-efficacy is demonstrated by the fact that our results show that students lacking both types of support have significant less efficacious beliefs.

In addition to the support of parents and teachers, specific features of the school environment can be conducive for academic self-efficacy [44,45]. We found evidences that the quality of school infrastructure, extracurricular activities, and the opportunity for personal abilities and talents development are positively associated with more efficacious beliefs. Activities and approaches that provide students with satisfactory experiences at school are those that can support self-efficacy. Such school environments enhance the self-confidence and satisfaction of students, improving self-efficacy, which will lead subsequently to better academic performances.

In urban areas, parents’ support in interaction with school environments characterized by the care for students’ abilities and talents and by infrastructure of quality very much improves the probability of high self-efficacy. On the other hand, lack of support from parents and teachers combined with poor-quality school infrastructure is associated with lowest probability of high self-efficacy. In rural areas, it is most probable that students lacking their parents support and who are not happy with their high school or educational profile will believe that they are not capable to pass the baccalaureate exam. As a consequence, they will be less motivated to study for the exam and less perseverant in their efforts. For rural students, predictors related to the school environment, including the teachers’ support, have no explicative power for self-efficacy. Thus, for students from rural areas, we found no interaction between features of the school environment and self-efficacy. This could be an indication of the need of investments in rural education institutions.

Furthermore, for some of the students, feelings regarding the extent in which the high school and educational profile in which they are currently enrolled in match their preferences and aspirations are important for the construction of self-efficacy. Students unsatisfied with the outcome of the previous exam and who feel that they are in the “wrong place” do not believe, to the same extent, in their capability to obtain a good result at the baccalaureate exam, which can affect their motivation and effort for entering into higher education and, subsequently, their entire life course. This result is in line with findings of previous studies [23,26]. In this respect, counselling services, which are underdeveloped in Romania, are very important for supporting educational choices, as well as resilience of students.

Differences of self-efficacy between students enrolled in general and vocational education are consistent with the fact that in some educational systems, including the one from Romania, vocational education is considered an educational path for students with low academic performances, available for those who fail to be admitted in general education. It seems that such views have been internalized by many students from vocational education programs. Also, confirming the results of previous studies [17,18], we found that gender differences in self-efficacy are more present in specific educational settings such as the vocational programs. Moreover, we found that in the case of the vocational track, the positive influence of characteristics of the school environment (satisfactory extracurricular activities and the opportunity for personal abilities and talents development) on self-efficacy is significantly higher among female students.

Our analysis has identified specific sub-groups of students who have the highest probability of failure beliefs at the baccalaureate exam, such as rural students lacking the parents support and who are not happy with the high school or educational profile in which they are currently enrolled in. Such students are most exposed to test anxiety, poor motivation, depression, or giving up to study for the exam. In fact, rural students are the only sub-sample for which feelings towards the high school and educational profile they are currently enrolled in shape self-efficacy. This could be an indication of the fact that the role of previous challenging similar experiences becomes significant for self-efficacy in the case of students lacking other types of resources (support of parents) and who are in school environments that are not conducive for self-efficacy.

The findings of our study argue that the perceived academic self-efficacy is a component of mental health and well-being of students that is embedded in the wider social and educational context. It is linked to the capabilities of families of the students, as well as with conditions of the educational environment which are being shaped by features of the education system and sub-systems.

For the future, authors aim to extend the study by analyzing the role of perceived self-efficacy in the baccalaureate exam as a mediator of the effects of socio-economic background and features of educational environment on the transition into higher education.

## 6. Conclusions

Our article has highlighted patterns of manifestation of perceived self-efficacy in relation with the baccalaureate exam among high school students in Romania. The results indicate significant relations between academic self-efficacy and perception of parents’ support, teachers’ support, as well as features of the school environment. Also, our analysis has identified specific sub-groups of students with higher prevalence of failure beliefs and vulnerability to test anxiety and depression. Our findings can be useful for building more resilient educational environments that support mental health and academic well-being of students from upper secondary education.

## Figures and Tables

**Table 1:** Distribution of the sample of students.

Characteristics		No.	%
Gender	Females	260	51.8
	Males	242	48.2
Educational Profile	General	272	54.2
	Vocational	230	45.8
Area of residence	Urban	302	60.2
	Rural	200	39.8
Subjective economic well-being of the origin household	Very good	49	9.8
	Good	125	24.9
	Pretty good	159	31.7
	Some difficulties	126	25.1
	With difficulties	27	5.4
	With lots of difficulties	16	3.2

Source: Authors’ calculation.

## Appendix A

**Table A1:** Statistics for independent variables included in the analysis.

Variables		%	Bootstrap for Percent			
			Bias	SE	95% Confidence Interval	
					Lower	Upper
Support of parents	Yes	48.2%	0	2.1	47.8	56.0
	No	51.8%	0	2.1	44.0	52.2
Support of teachers	Yes	26.7%	−0.1	1.8	69.5	76.9
	No	73.3%	0.1	1.8	23.1	30.5
Satisfactory extracurricular activities	Less extent	45%	0	2.2	40.8	49.8
	High extent	55%	0	2.2	50.2	59.2
Buildings, facilities and sport infrastructure of quality	Less extent	46.4%	0.1	2.1	42.2	51.0
	High extent	53.6%	−0.1	2.1	49.0	57.8
Opportunity for the development of personal abilities and talents	Less extent	36.7%	0	2.2	32.3	40.6
	High extent	63.3%	0	2.2	59.4	67.7
Hypothetical educational choice	Same high school, same educational profile	72.5%	0	2.0	68.5	76.5
	Same high school, different educational profile	8.2%	0	1.2	6.0	10.8
	Different high school, same educational profile	5.2%	0	1.0	3.4	7.2
	Different high school, different educational profile	14.1%	0	1.5	11.4	17.1

Note: Bootstrap results are based on 1000 stratified bootstrap samples. The bias indicates the magnitude of difference between the standard calculation and bootstrap version.
